# The Hidden Reserve of Nurses in The Netherlands: A Spatial Analysis

**DOI:** 10.3390/nursrep14020102

**Published:** 2024-05-28

**Authors:** Frits van Merode, Wim Groot, Catharina van Oostveen, Melline Somers

**Affiliations:** 1 Care and Public Health Research Institute, Maastricht University, P.O. Box 616, 6200 MD Maastricht, The Netherlands; w.groot@maastrichtuniversity.nl; 2 Maastricht University Medical Centre+, P. Debyelaan 25, 6229 HX Maastricht, The Netherlands; 3 Maastricht Graduate School of Governance, Maastricht University, Boschstraat 24, 6211 AX Maastricht, The Netherlands; 4 Erasmus School of Health Policy & Management, Erasmus University, Burgemeester Oudlaan 50, 3062 PA Rotterdam, The Netherlands; vanoostveen@eshpm.eur.nl; 5 Spaarne Gasthuis Academy, Spaarne Gasthuis Hospital, Spaarnepoort 1, 2134 TM Hoofddorp, The Netherlands; 6 Research Centre for Education and the Labour Market, Maastricht University, Tongersestraat 49, 6211 LM Maastricht, The Netherlands; melline.somers@maastrichtuniversity.nl

**Keywords:** nurse shortages, hidden reserve, regional differences, nurse mobility

## Abstract

Worldwide, nurse shortages constitute a problem, including in the Netherlands. Every region in the country has a shortage of all types of nurses. At the same time, there are large hidden reserves: persons who have been trained as a nurse but do not work in the healthcare sector. The size of the hidden reserve exceeds the shortage of nurses. Until now, the literature has not paid much attention to spatial aspects of the nursing shortage problem. In this paper, we analyze the size of the hidden reserves and how they are distributed over the country, across large and smaller cities, and across different nurse categories. We find that especially densely populated areas have relatively small shares of trained nurses as well as large hidden reserves relative to their population. These areas are also facing the largest nurse shortages. As nurse labor markets are local, policies that are more focused on local situations are necessary to activate these hidden reserves.

## 1. Introduction

Like many other countries, the Netherlands faces an increasing shortage of nurses. There are many reasons for this discrepancy between the supply and demand for nurses. One important reason is that nurses frequently leave the profession to work in another profession or industry. This creates a hidden reserve of nurses, i.e., workers with a diploma as a (registered) nurse who are not employed as a nurse or are not working in the healthcare sector. This hidden reserve can take different forms. It includes workers with a nursing diploma who are employed in another occupation in another industry, those who work fewer hours as a nurse than their full potential (part-time workers), and those who are not employed either because they are unemployed or out of the labor force (for example because they are in education). In the Netherlands, this hidden reserve of nurses is substantial [1]. Employment opportunities may vary between regions resulting in differences in pull factors that may tempt nurses to switch to a different industry or profession. The effect of push factors (for example job conditions in healthcare that are considered poor by nurses) interact with pull factors. If both factors are strong, the tendency to leave the healthcare sector will be large. Furthermore, regional differences in cost of living, mostly due to differences in housing prices, may cause nurses to be less likely to reside in some high-cost areas. Consequently, regional differences in the size of the hidden reserve of nurses may emerge.

Prior research has shown that the labor market of nurses is indeed very local [2,3,4]. According to Kovner et al. [4], both mobility and commuting among nurses are limited. The area of living, school, and the prospective workplace are typically within the same geographical area and at distances that are commutable [3,4]. These observations entail important policy consequences for training, attracting, and retaining nurses. Kovner et al. [4] emphasize the critical role of geographic positioning and the capacity of nursing schools. Similarly, Dahal et al. [3] argue that gaining insight into the distribution of nurses and their residential patterns is crucial for effective policymaking and strategic workforce planning.

There is limited evidence on the magnitude and the regional variance in the hidden reserve of nurses. Most of the evidence focuses on the available reserve of military and civilian nurses [5], emergency nurses [6], and on the potential of migrant nurses [7]. One study using a survey of registered nurses in the US found that in 2017, 61% of the registered nurses worked full-time as a nurse, while 17.3% were not working in nursing [8]. This study also found geographical variation in the hidden reserve of nurses, with more nurses working in other professions in more populous states.

In the Netherlands, the projected shortage of bachelor-trained nurses for 2023 was estimated to range between 5800 and 6000 full-time equivalents (fte) [9]. For vocationally trained nurses, the estimated shortage was between 8300 and 8700 fte, and between 9700 and 10,100 fte for nurse assistants [9]. Somers et al. (2023) found that the hidden reserve of trained nurses who do not work in the healthcare sector exceeds these shortages. For bachelor-trained nurses, the hidden reserve comprises more than 15,000 persons (20.3 percent of all trained nurses), for vocationally trained nurses almost 9000 persons (14 percent), and more than 16,000 (17.4 percent) for nurse assistants [1]. The hidden reserve of part-time working nurses is also substantial and exceeds the total shortage. Increasing all part-time contracts to full-time (36 h in the Netherlands) would result in 11,000 fte for both bachelor- and vocational-trained nurses and 22,000 fte for nurse assistants [1].

One of the reasons for this large hidden reserve could be the perceived working conditions in nursing occupations. In 2013, Aiken et al. [10] published a study on the working conditions and hospital quality of care in 12 countries in Europe, including the Netherlands. Nurses in all countries report that they are dissatisfied with important working conditions and many of them consider leaving their jobs [10]. However, significant variations exist between countries. The Netherlands has a relatively low percentage of dissatisfied nurses [10]. Similar to nurses in other countries, Dutch nurses experience limited opportunities for professional development, strained relations with management, and a lack of nurse leadership at the top of healthcare organizations [10]. Although the staff mix and the nurse-to-patient ratios vary substantially between countries, nurses in all countries are dissatisfied with their work conditions and the consequences this has for the quality of patient care [10]. Work conditions also vary within countries. Previous research in the Netherlands, for example, shows differences in autonomy and social support between intensive and non-intensive care nurses [11]. Moreover, this variation between non-intensive and intensive care departments within hospitals is larger than the variation between intensive care departments of different hospitals. Variations may also occur in other healthcare sectors. For example, specialized (diabetes) nurses experience relatively high levels of autonomy compared to nurses in other healthcare settings [12]. Variations may also exist within a country between regions. Dissatisfaction about work conditions appears to be a structural problem as a recent study shows that the work conditions have not changed very much [13].

In this study, we are especially interested in differences in the volume and composition of hidden reserves across regions. Available capacity influences work conditions and quality of care both directly and indirectly. The direct influence concerns the capacity available to get the “work done”. Indirectly, it concerns the amount of slack available to coordinate activities by the nurses themselves and to absorb uncertainty about the amount of work [14,15]. 

In this study, we analyze the extent of spatial variations in hidden reserves across different categories of nurses in the Netherlands. If geographic disparities in hidden reserves are present, it is important to understand these differences. Policies aimed at incentivizing nurses to re-enter or remain in the healthcare sector need to take the regional context into account. Implementing regionally tailored policies alongside national initiatives is essential for mitigating nurse shortages. We focus on the hidden reserve in the Netherlands and examine how different push and pull factors relate to the spatial distribution of the hidden reserve. We define the hidden reserve of nurses as the number of nurses who are trained as a nurse, nurse assistant, or healthcare assistant, but are not employed in the healthcare sector (anymore). We use registry data from the Netherlands to estimate the size of the hidden reserve at the municipality level in the Netherlands. The Dutch registry data distinguishes between four types of nursing degrees:

1. Bachelor-trained nurse (BN), EQF level 6;
2. Vocational-trained nurse (VN), EQF level 4;
3. Nurse assistant (NA), EQF level 3;
4. Healthcare assistant (HA), EQF level 2.

(The Dutch program names are: (1). hbo verpleegkundige, (2). mbo verpleegkundige, (3). verzorgende mbo niveau 3, (4). helpende mbo niveau 2.) 

EQF levels refer to the European Qualification Framework (EQF), established by the Bologna Process to ensure transparency and transferability of qualifications in Europe [16]. In the Netherlands, there are no nurses registered at the EQF levels 1 and 5. EQF level 1 refers to workers who support a patient and/or have experience as a patient. In The Netherlands, these support staff are not considered healthcare professionals. EQF level 5 nurse degrees do not exist in the Netherlands, although nurses are offered courses where they in fact attain this level. However, to manage the complexity of the national nurse function system from which the salary scales are derived the EQF level 5 is not a formally distinguished level. However, employers have the flexibility to reward nurses who succeed in obtaining extra diplomas on top of what is formally required for a certain level. In the Dutch case, and in our study, this means that EQF 5 nurses are included as EQF 4.

We describe how the size of the hidden reserve within municipalities or regions relates to different push and pull factors. Pull factors attract nurses to other occupations or to work as a nurse outside their own municipality or region. We expect that more outside career options increase the likelihood that individuals choose professions outside nursing. Pull factors related to outside career options include the availability of jobs with higher salaries or better career prospects. Pull factors also include circumstances associated with big city problems, like high housing costs. As proxies for these pull factors, we use the population size, the average income level of municipalities, and the distance to a region with a shortage of nurses. High hidden reserves and low shares of nurses in the population signal the effect of pull factors.

Push factors include nurse- and/or employer-related work, salary, and career conditions. If these are not considered favorable, these factors “push” nurses out of their jobs or out of healthcare. Even if nurses do not leave healthcare, push factors result in high turnover costs if nurses frequently change employers, especially in big cities [2]. A high turnover can be considered as a symptom that work conditions for nurses are dissatisfying [2].

This paper is structured as follows. First, we discuss the data and methods that we use to describe the spatial characteristics of trained nurses. Next, we present national-level results and figures at the municipal level. Given that many nurses work in municipalities different from where they reside but within a commutable distance, we also describe the regional situation. Specifically, we focus on the three largest cities in the Netherlands (Amsterdam, The Hague, and Rotterdam) and their surrounding areas, defined by the commutable distance. In the discussion section, we interpret the results and address the limitations of our methodological approach.

## 2. Data and Methods

### 2.1. Unit of Measurement

We analyze hidden reserves in the Netherlands at three levels: the country, the municipality, and the region. First, we describe the general characteristics of the hidden reserve of each professional group and how they relate to other characteristics, such as the share of the professional group relative to the total population. In all analyses, the unit of measurement is the municipality, as this is the smallest spatial unit available, due to privacy concerns. Second, we analyze differences between different types of municipalities. Municipalities differ in their characteristics, and although nurses frequently work in the neighborhood where they live, they often work in another municipality.

### 2.2. Registry Data Statistics Netherlands: Hidden Reserve

We use registry data from Statistics Netherlands (in Dutch: CBS) to calculate the hidden reserve of nurses at the end of 2021. The registry contains information about bachelor-trained nurses who received their diplomas between 2000 and 2021 and about vocationally trained nurses, nurse assistants, and healthcare assistants who obtained their diplomas between 2004 and 2021. For each individual, we observe whether they are employed in the healthcare sector or not. This group includes both salaried employees as well as self-employed workers. Individuals who are not employed in the healthcare sector either work in a different sector, are not employed, or are in school. We define the group of individuals who are not employed in the healthcare sector as the hidden reserve. In the registry data, we also observe in which municipality individuals reside. As the registration started in 2000, we have data from a considerable number of years that enable us to provide a very representative insight into the spatial characteristics of nurses, but it does not cover the nurses who received their nursing degree/diploma before 2000. Therefore, our findings underestimate the total hidden reserve. Nevertheless, it presents a picture of the available workforce to reduce the shortage of nurses in the labor market.

All hidden reserve data (total persons by nurse category, working in or outside healthcare) are available at the municipality level. When a municipality has fewer than 10 persons trained in a certain nursing profession, no data about that specific profession were provided, to secure the anonymity of persons living in small communities. We calculated the hidden reserve on 31 December 2021, because more recent data were not yet available when we conducted the study. For each of the 345 municipalities, we calculated the number of individuals who have been trained for one of the healthcare professions of interest. This resulted in 1380 municipality–nurse profession combinations. However, for 120 municipality–profession combinations, the number of trained individuals was below 10 and thus not included in our dataset. Table 1 provides an overview of the labor market status of all the trained nurses included in our study. The municipality of residence of 20,212 nurses could not be identified. The composition of this group is shown in Table A1 of Appendix A.

### 2.3. Open Data Statistics Netherlands: Population Size and Income Levels in Municipalities

The name and composition of municipalities sometimes change. We used the names and compositions of municipalities as of 1 January 2023. The names of the municipalities were obtained from the open data source of Statistics Netherlands [17]. The same open data were used to obtain information on the number of inhabitants of each municipality. As a proxy for the prosperity of the municipality, we used the mean individual income obtained from the open data source of Statistics Netherlands [18]. The income data in this file refer to 2020 as more recent data were not available. We assume that no substantial changes in the relative income levels between municipalities have taken place since then.

**Figure 1:**
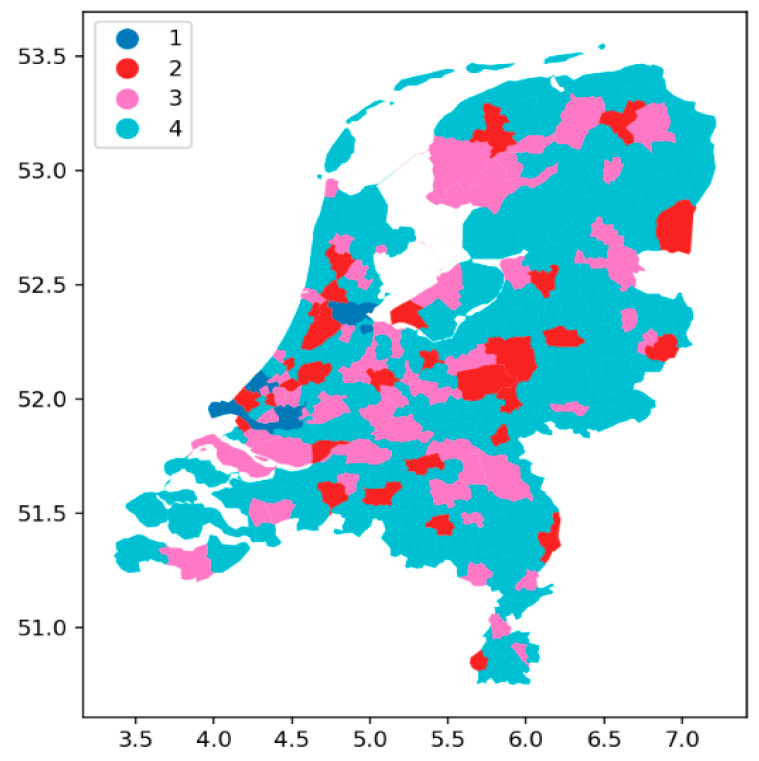
The geographical position of tier 1, 2, 3, and 4 municipalities (legend on the upper left of the map of the Netherlands). The x-axis shows the latitude, and the y-axis the longitude.

In urban geography [19], cities fulfill functions that depend on their size, geographic location, and relation with other cities and villages which fulfill functions at a certain distance. Although municipalities and cities are not the same in the Netherlands, there is a substantial overlap between big cities and the municipalities they belong to. The size of a city is an indication of its function, which could for example include offering 2nd or 3rd tier hospital care, providing higher education, and accommodating technology firms. In big cities, not only the population size is larger, but also the number of different functions, as well as the competition for personnel in tight labor markets. Table 2 shows how we classified Dutch municipalities into different categories according to their population size. Figure 1 depicts the geographical position of the tier 1, 2, 3, and 4 municipalities.

### 2.4. Regional Analyses

Regions in our analysis are defined using the COROP (COROP: Dutch: “Coördinatiecommissie Regionaal Onderzoeksprogramma”) region definition, which is used for statistical analyses and equivalent to the EU NUTS 3 region definition (NUTS: French: “Nomenclature des unités territoriales statistiques”). This regional definition enables us to observe differences at the national level and relate characteristics at the municipality level to the regional level. As an example, we choose the region of the biggest city in the Netherlands, Amsterdam. Next, we define regions as all the municipalities within a 35 km distance from the center of a certain municipality. We used linear regression to estimate the relation between the hidden reserve of the central municipality and the reserves of the surrounding municipalities. The dependent variable is the distance. We calculate for each regression model intercept, slope, and r^2^ for the relation between the distance and hidden reserve.

### 2.5. Software

All analyses have been performed with Python 3.8.6 [20]. In our Python programs, we used modules from the following packages: Pandas 2.2.0 [21,22], Matplotlib 3.7.0 [23,24], Seaborn 0.12.2 [25], NumPy 1.26.4 [26], and Geopandas 0.14.3 [27]. Latitude and longitude data were retrieved by GeoPy 2.4.1 [28]. The latitude and longitude data were also used to define the distances between municipalities. We used them also for the maps we present. In all maps, we present the latitudes at x-axis, and the longitude at the y-axis.

## 3. Results

### 3.1. Hidden Reserve at the Municipality Level

The mean, median, and standard deviation of the hidden reserve of all categories of nurses vary between the municipalities. This means that the distribution of the hidden reserves differs across municipalities (Table 3). The mean hidden reserve is on average highest among healthcare assistants (56.72% of trained healthcare assistants are not employed in the healthcare sector) and lowest among nurse assistants (17.76%).

**Figure 2:**
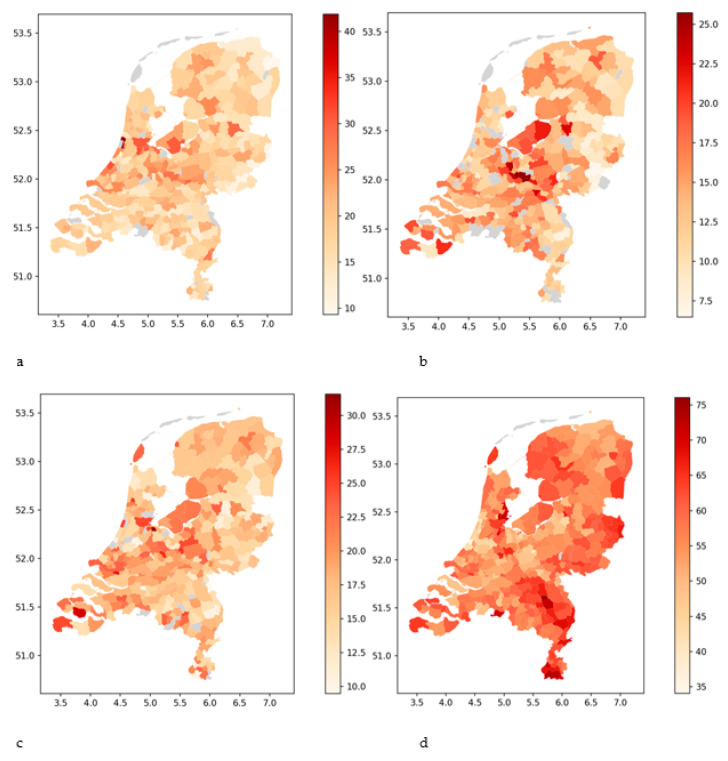
Hidden reserves of, respectively, bachelor-trained nurses (**a**), vocationally trained nurses (**b**), nurse assistants (**c**), and healthcare assistants (**d**). The hidden reserves are measured as percentages of the total number of persons trained for a specific nurse profession in a municipality. Grey areas indicate municipalities with less than 10 nurses of a certain category or that no data available were available. The x-axis shows the latitude, and the y-axis the longitude.

As shown in Figure 2, the hidden reserves of nurse professions vary significantly between municipalities. The hidden reserves of all nurse categories (with the exception of healthcare assistants) are highest in the center and in the metropolitan west of the Netherlands. The largest hidden reserves can be found in tier 1 municipalities, but also tier 2 and 3 municipalities have substantial hidden reserves. The distribution of hidden reserves of healthcare assistants differs from the other nurse categories.

#### 3.1.1. Share of Trained Nurses Relative to the Population at the Municipality Level

**Figure 3:**
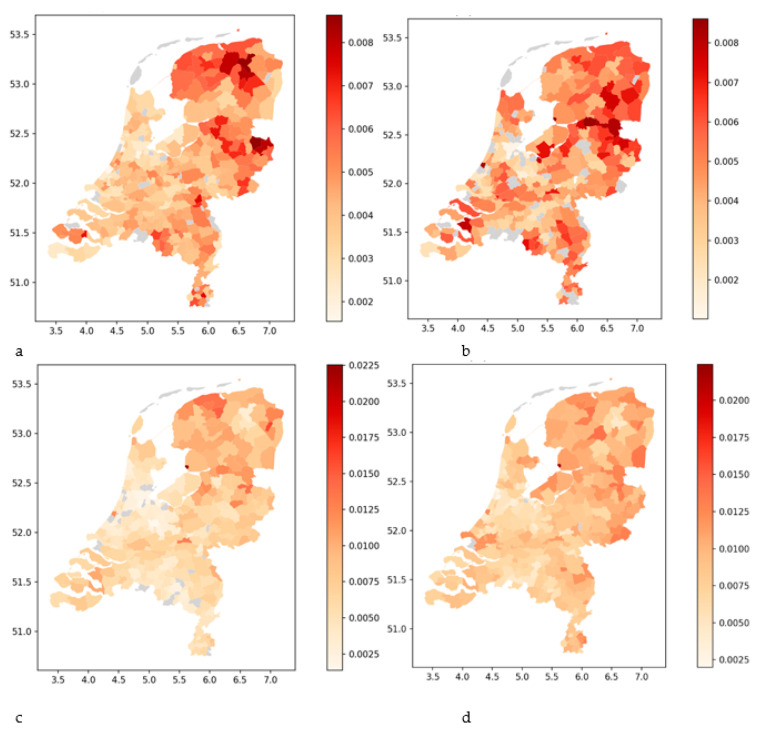
Shares of persons trained as nurses (hidden reserve and active) of the total population: bachelor-trained nurses (**a**), vocationally trained nurses (**b**), nurse assistants (**c**), and healthcare assistants (**d**). The shares are measured as a fraction from 0 to 1. The x-axis shows the latitude, and the y-axis the longitude.

Table 4 shows the share of individuals who are trained as a nurse (hidden reserve and active in the healthcare sector) relative to the total population. Table 4 illustrates that as the population size increases, the proportion of trained nurses relative to the population decreases. This especially holds for the tier 1 municipalities. At the same time, we observe that in tier 1 areas, a relatively large share of nurses have left the healthcare sector (i.e., the hidden reserves are large). For 2nd and 3rd tier municipalities, this relationship is weaker, and the location of the region becomes dominant (see Figure 3). Especially the northeast of the Netherlands has a relatively large share of the population trained as a nurse. The region is dominated by tier 4 municipalities, but with some 2nd and 3rd tier municipalities.

The maps of the Netherlands in Figure 3 below show the spatial distribution of the share of persons trained as nurses (relative to the population) at the municipality level. Note that the actual shares are much higher as we only have data available from 2000 to 2021 for bachelor-trained nurses and from 2004 to 2021 for the other categories of nurses. 

#### 3.1.2. Hidden Reserves Related to Personal Income at the Municipality Level

When we compare the average individual income of municipalities with the share of individuals trained as a nurse, we observe that an increase in the average individual income of municipalities is associated with a decrease in the share of persons trained as a nurse (Table 5). The strength of this relation increases as the educational level of the nurses decreases. Moreover, our findings indicate a positive correlation between the average individual income and the size of hidden reserves at the municipal level. Municipalities with a higher average individual income have fewer practicing nurses living in their municipality. This particularly holds for nurses with lower levels of education.

#### 3.1.3. Dependencies between Hidden Reserves

Tasks of different categories of nurse professions may overlap in practice. As a consequence, healthcare organizations can choose to let bachelor-trained nurses also perform the tasks of vocationally trained nurses, which could cause a dependency between the hidden reserves of these professional nursing groups. Nevertheless, the hidden reserves of bachelor-trained and vocational-trained nurses seem complementary to each other. The same holds for nurse assistants and vocational and bachelor-trained nurses. The hidden reserve of healthcare assistants does not seem to have any relationship with the other groups. Overall, if there are correlations then these are mostly positive and moderate (Table 6). As the nursing shortages are large across the country, strong substitution effects are not to be expected.

### 3.2. Hidden Reserves at the Regional Level

Shifting the focus from the municipality level to the regional level might explain differences between municipalities, as observed above, since nurses might work in a municipality different from where they live. Moreover, a focus on the regional level may give more insight into the segregation between living and working areas. 

For the regional level, we focus on the largest municipalities of tier 1: Amsterdam, The Hague, and Rotterdam. All three cities are part of the metropolitan area “Randstad” and are situated within 100 km of the others. Municipalities of tier 2 are situated between all three cities. To analyze the environment of the tier 1 municipalities, we consider the areas within 35 km from the center of each of the three cities. 

#### 3.2.1. Amsterdam Region

**Figure 4:**
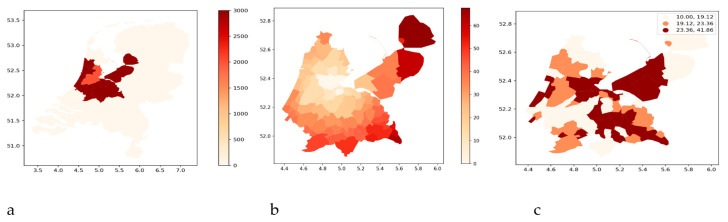
(**a**) The position of the Amsterdam COROP area with the surrounding COROP areas within the Netherlands. (**b**) The distances in km of the municipalities of these COROP areas from the center of Amsterdam. (**c**) The three quintiles (1/3 and 2/3) of the hidden reserve of bachelor nurses (see the legend on the top right of the map). The x-axis shows the latitude, and the y-axis the longitude.

To explain our method of analysis, we discuss the largest city in the Netherlands (Amsterdam) and its surroundings more extensively. We include the COROP area in Amsterdam and the COROP areas adjacent to its borders in the analyses. These COROP areas are 17, 19, 20, 21, 22, 23, 24, 25, 28, and 40 as indicated in Figure 4.

**Figure 5:**
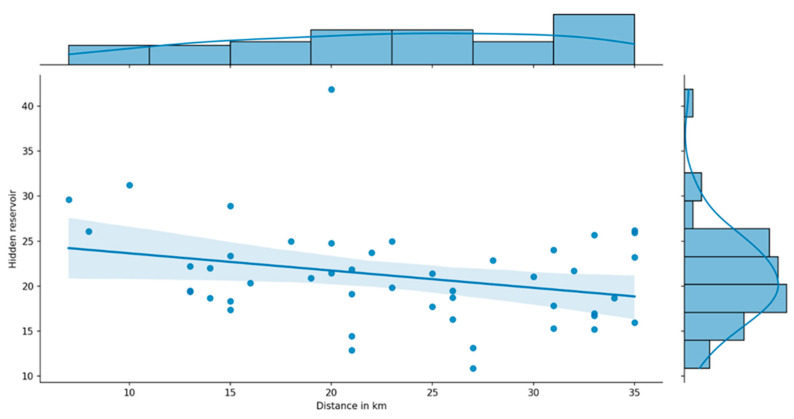
The relation between the distance of municipalities in km from Amsterdam and the hidden reserves among bachelor-trained nurses of municipalities within 35 km. Dots indicate municipalities. The line between them is based on linear regression analysis.

From Figure 4c, we can observe that as the distance from Amsterdam increases, the hidden reserve (darker areas) diminishes. This is confirmed by Figure 5, which is based on linear regression analysis relating the distances of municipalities from Amsterdam to the hidden reserve.

#### 3.2.2. Amsterdam, the Hague and Rotterdam Regions

A distance effect is found for all nurse categories at the tier 1 municipalities, except for vocational-trained nurses (Table 7). When the distance increases, municipalities have smaller hidden reserves and a higher share of nurses relative to the population. Hence, tier 1 municipalities do not only have relatively smaller shares of nurses relative to their population compared to other municipalities in the neighborhood, but they also often do not work as nurses. Distance effects for bachelor-trained nurses at 2nd tier municipalities are shown in Table A2 in Appendix A. The relationship between the hidden reserve of these municipalities and the distance effect is less clear.

#### 3.2.3. All Regions in The Netherlands

**Figure 6:**
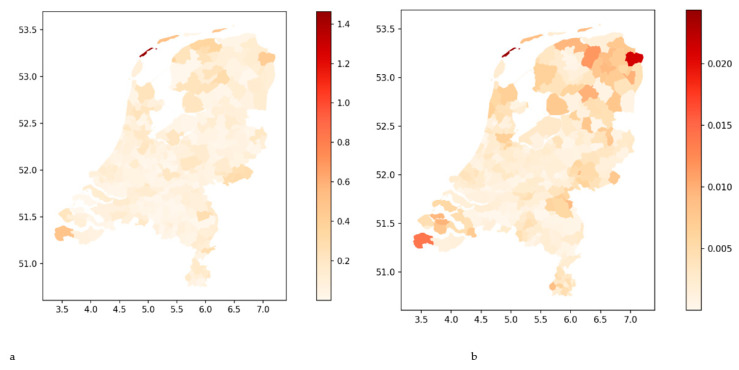
Maps of regional difference of hidden reserve (**a**) and share of the population (**b**) for bachelor nurses. For each municipality, a regression model (with intercept) is estimated with the distance between that municipality and all other municipalities within 35 km reach as independent variables and the hidden reserve (**a**) and share of the population (**b**) as dependent variables. The regression slopes are used to color the maps. The darker the area, the more differences exist between municipalities within that region. In other words, the lighter the area, the more homogenous the region. Note that the colors are relative. The same color at different places on the map cannot be compared. The x-axis shows the latitude, and the y-axis the longitude.

By extending the regression analyses for bachelor-trained nurses to all Dutch municipalities, we found that the regional effects are present everywhere (Figure 6), but also often with a low explained variance (R^2^). 

## 4. Discussion

Our analysis showed that there is a significant geographic variation in the size of hidden reserves and the share of trained nurses relative to the total population size of a municipality. In addition to the geographic variation, there is also variation across the different nurse categories. 

The western region of the Netherlands, including cities such as Amsterdam, The Hague, Rotterdam, and several 2nd tier municipalities, known as the “Randstad” or the Dutch metropolitan area, exhibits varying levels of hidden reserves across all nurse categories. However, predominantly among healthcare assistants, there are high hidden reserves. Despite being highly urbanized, this area has a low proportion of trained nurses relative to the total population, across all nurse categories. The only exception is some areas in the west, which have a medium proportion of vocationally trained nurses. In the Dutch metropolitan area, there is segregation between first-tier municipalities and several second-tier municipalities and their surroundings. The same phenomenon is visible in a number of 2nd tier municipalities outside the metropolitan west, but less prominently. This suggests that the 1st tier and some 2nd tier municipalities depend on nurses from surrounding municipalities which have higher shares of trained nurses relative to the population compared to the 1st tier municipalities, but lower compared to the rest of the Netherlands. Because nurse labor markets are local [2,3,4], longer commuting distances are unlikely. Pulling nurses to metropolitan areas from surrounding areas would likely require significant incentives.

Our findings also clearly show a negative association between the hidden reserve and the distance from the metropolitan centers. The hidden reserves are larger within metropolitan areas than outside them. Furthermore, we observe a negative association between the hidden reserve and the mean individual income in an area. These findings suggest that individuals with a nurse qualification, who earn an average income, are more likely to be pushed out of the metropolitan areas where the cost of living—and especially the cost of housing—is higher. Workers with a nurse qualification who remain in the metropolitan area are more inclined to leave the nursing profession for better-paying jobs in other industries, thereby contributing to the hidden reserve. Additionally, the relatively smaller hidden reserve outside metropolitan areas suggests that it may be more challenging to incentivize nurses from these areas to relocate to metropolitan areas with higher living costs.

Our findings raise questions about the feasibility of policies aimed at encouraging nurses to reside and work, particularly in first municipalities. The spatial associations presented in this paper do not permit causal inferences or definitive conclusions about the push and pull factors that may explain them. Nurses may be pushed out of a healthcare organization, for example, because of work conditions. If there are numerous local opportunities to work in other healthcare organizations, turnover rates will increase, resulting in higher concentrations of nurses in these local and nearby organizations. This will lead to extra costs for organizations in metropolitan areas to attract nurses and retain them. Alternatively, nurses may choose to leave the healthcare sector and seek employment in other industries. Notably, the hidden reserve of bachelor-trained nurses in the three major cities is significant compared to the surrounding municipalities. As all these municipalities are part of a metropolitan area, and if working as a nurse in a big city is considered less attractive, nurses have enough alternatives to work in the surrounding cities. When there are not many options to work in healthcare locally, push factors will result in nurses leaving the healthcare sector or opting out by an accumulation of dissatisfaction, stress, and sickness absence. Hence, the effects of push factors may vary between metropolitan and rural areas. 

However, do the big cities have the same push and pull factors? The hidden reserves, especially those of bachelor-trained nurses and healthcare assistants, differ between the three major cities. It is challenging to speculate about the differences in push factors between these cities based on the existing literature and our findings. However, insights from the urban geography literature suggest that pull factors are closely tied to the size and function of a city, where the number and variety of job opportunities generally increase with the size of the city [19]. This is also the rationale for dividing Dutch municipalities into a hierarchy of four tiers, with tier 1 cities offering not only more jobs but also a greater diversity of job types. The number of job types in these cities increases the likelihood of attracting nurses to alternative employment opportunities, potentially explaining the differences in hidden reserves between the three major cities and the rest of the Netherlands. Nonetheless, this explanation falls short of explaining the differences among these three cities, unless we delve deeper into their specific functions and job markets.

In addition to job opportunities, the three cities also differ in other factors, especially in average personal income and their relationship with hidden reserves in the surrounding municipalities. According to data from Statistics Netherlands, the average personal incomes (in 2020) vary substantially and are as follows: Amsterdam: EUR 32,400, The Hague: EUR 27,800, and Rotterdam: EUR 26,000 [18]. The Pearson correlation coefficients presented in Table 4 indicate that these differences align with variations in the hidden reserves for nursing professions. However, it is important to interpret these patterns with caution as these correlations are based on data from all municipalities. 

Apart from average personal income, differences in hidden reserves between Tier 1 cities and their surrounding municipalities are also evident. This is reflected in the distance slopes shown in Table 7. Amsterdam exhibits distance slopes for bachelor nurses that are more than twice as large as those of Rotterdam and The Hague. For healthcare assistants the patterns are different (Table 7), Amsterdam has the highest hidden reserve with substantially lower hidden reserves in the surrounding municipalities. Rotterdam shows a similar trend, albeit to a lesser degree, while The Hague’s hidden reserve is relatively low, with a slope of almost 0. Considering the small differences in hidden reserves of healthcare assistants (Table 4) between tiers of municipalities, significant big city effects are unlikely. The largest big city effects are observed for bachelor-trained nurses, whose proportion relative to the total nursing staff is considered a significant contributor to the quality of care. Therefore, stimulating the bachelor-trained hidden reserve is of utmost importance.

Pull factors are attractive options outside the healthcare sector. Especially working conditions, salaries, and career prospects in other economic sectors might lead nurses to decide to work elsewhere. These pull factors will especially operate in metropolitan areas where employment opportunities are high and there are enough alternative jobs. In rural areas with fewer options to work in other healthcare organizations or other economic sectors, pull factors are probably less important.

The fact that the labor market of nurses is local also has consequences for the distinction between rural and urban areas. In the Netherlands, the distinction between rural and non-rural is relative, as it is a densely populated country. Consequently, there are 2nd, 3rd or even 4th tier cities in close proximity to metropolitan areas with economic sectors that are attractive for nurses. 

The shortages are substantial in all regions for all professional groups, although the extent of the shortage differs between regions. The shortages may affect the size of the hidden reserve, but as there is a general shortage, we do not expect it to influence the distribution of the hidden reserves. This could be different if there were regions or professional groups with no shortages. Given the local nature of nurse labor markets, there is an even greater imperative to design policies targeting particular regions. For example, giving priority to assigning houses to essential professionals such as nurses in big cities, paying a regional bonus, and extending the nursing school capacity in areas with high shortages. Based on our findings, we also expect that workers in major cities are more sensitive to wage increases than nurses in other areas in the Netherlands. Healthcare organizations in major cities could also attract (former) nurses from surrounding municipalities by offering sufficient financial compensation for longer commuting times. 

More generally, activating the hidden reserve could involve strategies such as reducing work pressure and providing greater control over working hours, salary, and autonomy. Many healthcare organizations provide training programs to support former nurses in returning to the profession and renewing their nurse qualification, which may expire if they have been out of practice for a certain period. It is important to note that the hidden reserve of nurses not only includes former nurses working in other sectors but also a significant portion of inactive workers who are currently unemployed [1]. Therefore, different groups of inactive nurses may require different incentives to re-enter the nursing profession. An important avenue for further research is to examine the effectiveness of different interventions in stimulating inactive nurses to re-enter the profession. 

Although all these measures to mitigate nurse shortages could be considered, evidence of their impact is lacking not only on the reduction in nurse shortages but also on the externalities. For example, giving priority to nurses when assigning affordable housing does not improve the push factors, but only reduces the pull of other sectors. As other sectors may also have problems with shortages of staff, this priority policy goes at the expense of these other industries. 

### Strengths and Limitations

To the best of our knowledge, this is the first study in the Netherlands about the size and composition of the hidden reserve of nurses analyzed from a spatial perspective. In general, the literature about hidden reserves is limited. However, registry data make it possible to analyze the regional differences in the hidden reserve. This is possibly also the case in other countries, and we suggest that more research should be conducted on the spatial aspects of the hidden reserves.

Although we think that our spatial analysis of the hidden reserve provides useful evidence, not all important regional aspects are captured. For instance, the Netherlands is surrounded by a lengthy border with Germany and Belgium. In some areas, Belgian nurses work in Dutch healthcare. In other areas, Dutch nurses work in German healthcare. The geographic distribution of the nurse workforce might be path-dependent and influenced by historical developments. For example, in the aftermath of the 2008 financial crisis, austerity policies within the public sector, including healthcare, restricted the inflow of nurses in nursing schools and health institutions. The impact of these measures may be long-lasting. As nurse education takes a few years, recovery from a temporary drop takes at least this time. Moreover, due to the deliberately caused shortage of nurses, push factors may have become stronger. The effect of these push factors also depends on the role of the municipalities. Our analyses are conducted at the level of municipalities. In urban geography, see, e.g., [19], these distinctions are often used to distinguish cities not only on the basis of their population size but also to specify their function in the region. Depending on the functions of the city, the regions may vary. A municipality is not the same as a city, but it may cover a region with numerous villages and small towns and be part of an agglomeration. For privacy reasons, we were restricted from performing our analyses at the municipality level and could not perform analyses at the village or town quarter level.

Another limitation of our study is that bachelor-trained nurses who obtained their last degree/diploma before 2000 are not included in this study. Other categories of nurses were excluded if they finished their nursing training before 2004. The number of trained nurses included in the analyses is high enough to be representative of the pattern of spatial distribution, but not of the absolute number. However, we do not know if older nurses (who graduated before 2000) have other mobility or commuting patterns.

Because we did not observe the actual occupation of trained nurses, we categorized all those employed within healthcare as actively contributing to the nursing field. Consequently, our study underestimates the true magnitude of the hidden reserve, as some individuals may hold positions in healthcare management or other related fields. This limitation notwithstanding, nurses engaged in health-related roles both within and beyond traditional healthcare settings play a vital role in shaping the quality and advancement of nursing practice. We recommend expanding nurse registries to encompass roles beyond the confines of the healthcare sector.

## 5. Conclusions

Significant hidden reserves exist for all categories of nurses across all regions. There are two types of heterogeneity visible: at the level of the country and between the big cities and the surrounding municipalities. Especially, the big cities have smaller shares of persons trained as a nurse and a larger hidden reserve at the same time. This suggests that the big cities have pull factors to attract nurses to other jobs, but also that persons living in big cities are less inclined to be trained as nurses. The differences in hidden reserves as a share of the population at the country level and within the regions have consequences for policies directed to stimulate nurses to stay or return to their jobs.

## Figures and Tables

**Table 1:** Overview of trained nurses included in our study. The column “Total” refers to the number of nurses who received their diploma between 2000 and 2021 (bachelor-trained) or 2004 and 2021 (vocational-trained, nurse assistant, healthcare assistant). “In healthcare” refers to the nurses working in the healthcare sector and “Out healthcare” refers to the nurses who work outside healthcare or are not registered as having a job.

	Total	In Healthcare		Out Healthcare	
Bachelor-trained nurse	13,023	10,773	83%	2250	17%
Vocationally trained nurse	11,121	9750	88%	1371	12%
Nurse assistant	41,869	34,812	83%	7057	17%
Healthcare assistant	119,622	52,476	44%	67,146	56%
Total	185,635	107,811	58%	77,824	42%

**Table 2:** The ordering of municipalities in this study. The three municipalities of category 1 are Amsterdam, The Hague, and Rotterdam.

Tier	Population Size	Number of Municipalities
1	>500,000	3
2	100,000–500,000	29
3	50,000–100,000	60
4	<50,000	250

**Table 3:** Hidden reserve variation between municipalities. The hidden reserves are measured as percentages of the total number of persons trained for a specific nurse profession in a municipality.

Profession	Hidden Reserve				
	Mean	Median	*sd*	*q1*	*q3*
Bachelor-trained nurse	18.97	18.68	4.42	16.99	20.61
Vocationally trained nurse	13.92	13.81	3.39	12.42	15.03
Nurse assistant	17.76	17.51	3.55	16.37	18.86
Healthcare assistant	56.72	56.88	6.44	53.95	59.49

**Table 4:** Hidden reserves and the share of trained nurses relative to the total population for the four tiers of municipalities.

		Hidden Reserve (%)			Share of Total Population (%)		
		Mean	Median	*sd*	Mean	Median	*sd*
Municipality Ranking	Profession						
1	Bachelor-trained nurse	27.76	26.93	2.37	0.246	0.237	0.022
	Vocationally trained nurse	17.07	17.23	0.76	0.195	0.211	0.066
	Nurse assistant	22.64	23.46	2.55	0.319	0.319	0.135
	Healthcare assistant	60.85	59.88	2.80	0.967	1.004	0.240
2	Bachelor-trained nurse	21.17	21.07	2.45	0.435	0.388	0.146
	Vocationally trained nurse	15.28	14.69	2.97	0.339	0.329	0.102
	Nurse assistant	18.68	18.69	2.78	0.448	0.444	0.178
	Healthcare assistant	57.56	56.12	5.22	0.788	0.769	0.204
3	Bachelor-trained nurse	19.45	18.63	4.21	0.406	0.390	0.113
	Vocationally trained nurse	14.21	14.13	3.00	0.429	0.415	0.145
	Nurse assistant	18.11	17.54	3.31	0.603	0.596	0.245
	Healthcare assistant	55.86	55.97	5.49	0.899	0.895	0.229
4	Bachelor-trained nurse	18.43	17.81	4.48	0.446	0.430	0.119
	Vocationally trained nurse	13.55	13.28	3.52	0.490	0.469	0.133
	Nurse assistant	17.44	17.21	3.62	0.669	0.618	0.275
	Healthcare assistant	56.79	57.23	6.81	0.833	0.826	0.254

**Table 5:** Pearson correlation between average personal income of municipalities and hidden reserves and share of trained nurses.

Profession	Average Personal Income of Municipality	
	Hidden Reserve	Share of Trained Nurses Relative to Population
Bachelor-trained nurse	0.40	−0.29
Vocationally trained nurse	0.27	−0.57
Nurse assistant	0.13	−0.76
Healthcare assistant	−0.21	−0.78

**Table 6:** Pearson correlation matrix of hidden reserves of professional groups (municipalities are compared).

Profession	Bachelor-Trained Nurse	Vocational-Trained Nurse	Nurse Assistant	Healthcare Assistant
Bachelor-trained nurse	1.0 ***	0.38 ***	0.32 ***	−0.14 *
Vocational-trained nurse	0.38 ***	1.0 ***	0.44 ***	−0.03
Nurse assistant	0.32 ***	0.44 ***	1.0 ***	0.04
Healthcare assistant	−0.14 *	−0.03	0.04	1.0 ***

*p*-values: * 0.05, *** 0.001.

**Table 7:** Distance effects of all nurse categories for tier 1 municipalities Amsterdam, The Hague, and Rotterdam.

Municipality	Intercept	Slope	R^2^
Bachelor-trained nurses			
Amsterdam	25.5760	−0.1920	0.0822
The Hague	21.9948	−0.0898	0.0342
Rotterdam	21.3827	−0.0891	0.0368
Vocational-trained nurses			
Amsterdam	12.5590	0.0463	0.0108
The Hague	14.3861	−0.0413	0.0163
Rotterdam	15.1892	−0.0848	0.0719
Nurse assistants			
Amsterdam	19.1239	−0.0680	0.0261
The Hague	18.5603	−0.0003	0.0000
Rotterdam	21.2363	−0.1358	0.1173
Healthcare assistant			
Amsterdam	60.6870	−0.3075	0.1167
The Hague	50.7542	0.0290	0.0016
Rotterdam	57.0969	−0.2342	0.1087

## Data Availability

Data sharing is not applicable. Data are obtained from Statistics Netherlands: https://www.cbs.nl.

## Public Involvement Statement

The advisory committee involved is composed of representatives of Dutch healthcare organizations and the Dutch Professional Association of Nurses (V&VN).

## Guidelines and Standards Statement

Registry-based study.

## Appendix A

**Table A1:** Nurses who were not included in our study as the municipality of residence could not be identified.

	Total	In Healthcare		Out Healthcare	
Bachelor-trained nurse	3233	1393	43%	1840	57%
Vocational-trained nurse	2589	1281	49%	1308	51%
Nurse assistant	3602	1466	41%	2136	59%
Healthcare assistant	10,788	1271	12%	9517	88%
Total	20,212	5411	27%	14,801	73%

**Table A2:** Distance Effects of 2nd Tier Cities for Bachelor-trained Nurses.

Municipality	Intercept	Slope	R^2^
Alkmaar, Netherlands	16.4355	0.2444	0.1244
Almere, Netherlands	24.0840	−0.0915	0.0165
Alphen aan den Rijn, Netherlands	19.8124	0.0577	0.0073
Amersfoort, Netherlands	21.4344	0.0155	0.0009
Apeldoorn, Netherlands	23.3646	−0.1468	0.0524
Arnhem, Netherlands	20.8309	−0.0601	0.0173
Breda, Netherlands	18.0696	−0.0438	0.0140
Delft, Netherlands	22.6407	−0.1330	0.0811
Deventer, Netherlands	17.7203	0.0129	0.0006
Dordrecht, Netherlands	16.4487	0.0943	0.0515
Ede, Netherlands	25.2019	−0.2014	0.1204
Eindhoven, Netherlands	17.5142	−0.0205	0.0023
Emmen, Netherlands	13.4753	0.1320	0.0533
Enschede, Netherlands	16.1238	−0.0125	0.0009
Groningen, Netherlands	17.2411	−0.1039	0.0606
Haarlem, Netherlands	24.0623	−0.1176	0.0279
Haarlemmermeer, Netherlands	23.6330	−0.0911	0.0171
Leeuwarden, Netherlands	18.8910	0.0107	0.0005
Leiden, Netherlands	20.0460	0.0527	0.0086
Maastricht, Netherlands	13.4833	0.1709	0.1590
Nijmegen, Netherlands	19.2035	−0.0016	0.0000
‘s-Hertogenbosch, Netherlands	16.6922	0.0639	0.0231
Tilburg, Netherlands	18.4988	−0.0386	0.0089
Utrecht, Netherlands	21.6376	−0.0121	0.0005
Venlo, Netherlands	21.2817	−0.1211	0.0489
Westland, Netherlands	23.8008	−0.1460	0.0883
Zaanstad, Netherlands	23.6118	−0.0965	0.0207
Zoetermeer, Netherlands	20.2263	0.0165	0.0008
Zwolle, Netherlands	15.9839	0.1369	0.0994
